# Completion of the modified World Health Organization (WHO) partograph during labour in public health institutions of Addis Ababa, Ethiopia

**DOI:** 10.1186/1742-4755-10-23

**Published:** 2013-04-18

**Authors:** Engida Yisma, Berhanu Dessalegn, Ayalew Astatkie, Nebreed Fesseha

**Affiliations:** 1 Department of Nursing, Faculty of Medical and Health Sciences, Samara University, Samara, Ethiopia; 2 Department of Nursing and Midwifery, School of Allied Health Sciences, Addis Ababa University, Addis Ababa, Ethiopia; 3 School of Public and Environmental Health, College of Medicine and Health Sciences, Hawassa University, Hawassa, Ethiopia; 4 JSI L10k Research and Training Institute, Addis Ababa, Ethiopia

**Keywords:** Modified WHO partograph, Completion, Public health institutions, Addis Ababa, Ethiopia

## Abstract

**Background:**

The World Health Organization (WHO) recommends using the partograph to follow labour and delivery, with the objective to improve health care and reduce maternal and foetal morbidity and death. The partograph consists of a graphic representation of labour and is an excellent visual resource to analyze cervix, uterine contraction and foetal presentation in relation to time. However, poor utilization of the partograph was found in the public health institutions which reflect poor monitoring of mothers in labour and/or poor pregnancy outcome.

**Methods:**

A retrospective document review was undertaken to assess the completion of the modified WHO partograph during labour in public health institutions of Addis Ababa, Ethiopia. A total of 420 of the modified WHO partographs used to monitor mothers in labour from five public health institutions that provide maternity care were reviewed. A structured checklist was used to gather the required data. The collected data were analyzed using SPSS version 16.0. Frequency distributions, cross-tabulations and a graph were used to describe the results of the study.

**Results:**

All facilities were using the modified WHO partograph. The correct completion of the partograph was very low. From 420 partographs reviewed across all the five health facilities, foetal heart rate was recorded into the recommended standard in 129(30.7%) of the partographs, while 138 (32.9%) of cervical dilatation and 87 (20.70%) of uterine contractions were recorded to the recommended standard. The study did not document descent of the presenting part in 353 (84%). Moulding in 364 (86.7%) of the partographs reviewed was not recorded. Documentation of state of the liquor was 113(26.9%), while the maternal blood pressure was recorded to standard only in 78(18.6%) of the partographs reviewed.

**Conclusions:**

This study showed a poor completion of the modified WHO partographs during labour in public health institutions of Addis Ababa, Ethiopia. The findings may reflect poor management of labour or simply inappropriate completion of the instrument and indicate the need for pre-service and periodic on-job training of health workers on the proper completion of the partograph. Regular supportive supervision, provision of guidelines and mandatory health facility policy are also needed in support of a collaborative effort to reduce maternal and perinatal deaths.

## Background

The first graphic assessment of progress of labour was designed by Friedman and further improved by Philpott and Castle [1]. Much work has been done to improve the partograph as a tool which graphically represents key events during labour and adapts it for use globally. In response to the recommendations of the Safe Motherhood Conference held in Nairobi in 1987, the WHO produced a partograph, and tested its practical value to reduce maternal and perinatal morbidity and mortality [2]. Partograph use is recommended for routine monitoring of labour, and helps the health care provider in identifying slow progress in labour, and may help initiate appropriate interventions to prevent prolonged and obstructed labour [3,4].

The partograph is an inexpensive tool designed to provide a continuous pictorial overview of labour and has been shown to improve outcomes when used to monitor and manage labour. It is a single sheet of paper which includes information about the foetus’ heart rate, uterine contraction, any drugs used and other important factors that could help avoid extensive descriptive notes. It is a practical device when employed in a busy labour room with many cases, but limited personnel to screen for abnormal labour. With its use, there is no need to record labour events repeatedly. It helps predict deviation from normal progress of labour, and supports timely and proven intervention. It also helps to facilitate responsibility to the person conducting labour [5].

**Figure 1:**
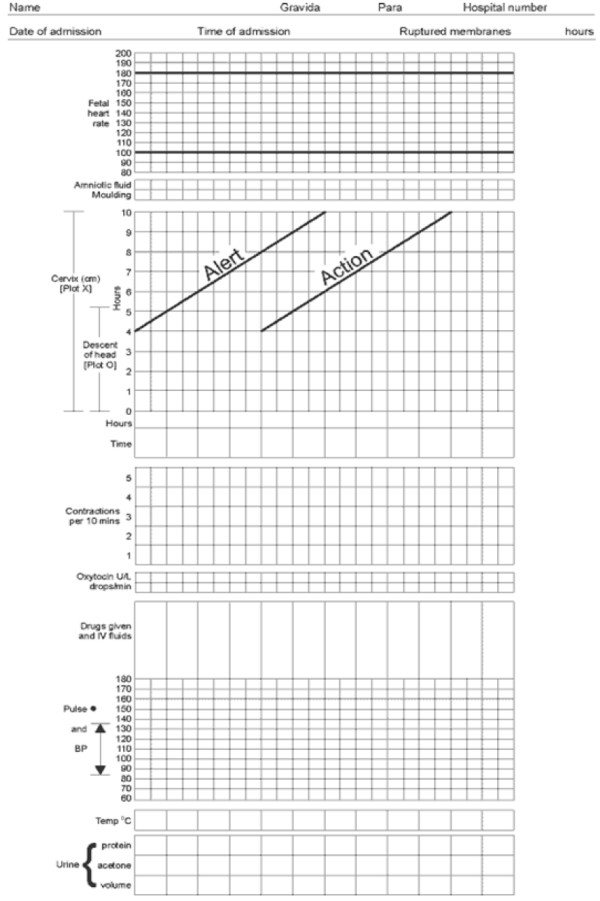
**The modified WHO paragraph **[7].

The first WHO partograph or ‘Composite partograph’, covers a latent phase of labour of up to 8 hours and an active phase beginning when the cervical dilatation reaches 3 cm. The active phase is depicted with an alert line and an action line, drawn 4 hours apart on the partograph. This partograph is based on the principle that during active labour, the rate of cervical dilation should not be slower than 1 cm/hour. Since a prolonged latent phase is relatively infrequent and not usually associated with poor perinatal outcome, the usefulness of recording the latent phase of labour in the partograph has been questioned. Moreover, differentiating the latent phase from false labour is often difficult [6]. To alleviate these disadvantages, a modified WHO ‘partograph’ (see Figure 1) was introduced and incorporated removal of the latent phase and defined the beginning of the active phase at 4 cm cervical dilatation instead of 3 cm [1].

A study conducted in Addis Ababa, Ethiopia on knowledge and utilization of partograph among obstetric care givers in public health institutions showed that over half (57.3%) of the obstetric care givers reported use of the modified WHO partograph to monitor mothers in labour [8]. The present study, which is intended to answer whether or not the partographs that are used to monitor mothers in labour are recorded to the standard via retrospective document review, aimed to assess correct completion of partographs for mothers in labour in public health institutions of Addis Ababa, Ethiopia. A fuller understanding of this process will be important to inform policies and strategies in the provision of maternity care services.

## Methods

### Study setting

The study was conducted from February 28, 2012 to March 30, 2012 in Addis Ababa, the capital city of Ethiopia and seat of African Union and the United Nations World Economic Commission for Africa. Addis Ababa has a population of over 3 million (3,038,096) with annual growth rate of 2.1% (data obtained from central statistical agency of Ethiopia) and is located at 9° 1′ 48″ North and 38° 44′ 24″ East with a total land area of 54,000 hectares. Its average elevation is 2,500 meters above sea level, and hence has a fairly favorable climate and moderate weather conditions.

The city has 48 hospitals. Thirteen are public hospitals of which, 5 are under Addis Ababa Regional Health Bureau (AARHB) jurisdiction and 5 are specialized referral (central) hospitals. Furthermore, the city has 32 health centers under the Addis Ababa Health Bureau. There are also two hospitals, three health centres and 31 clinics established by non-government organizations (NGOs), and 33 hospitals and more than 700 clinics that are privately owned.

### Study design

A descriptive study based on a retrospective document review was used to examine the completion of a modified WHO partograph during labour in public health institutions of Addis Ababa.

### Population

The source population comprised all the modified WHO partographs that had been used to monitor labour in public health institutions of Addis Ababa from December 2011—February 2012. Study subjects were comprised of a random sample of the modified WHO partographs.

### Inclusion and exclusion criteria

This study included all the modified WHO partographs having complete or partially complete information and excluded those modified WHO partographs which had no information recorded on them (such as partograph sheets on which only a delivery summary is recorded but no written evidence of dilatation of the cervix and/or records of mothers who were admitted in second stage of labour). In addition to this, mothers’ files having information showing prolonged labour, severe oligohydraminous, intrauterine foetal death (IUFD), previous caesarean section plus breach presentation, Human Immunodeficiency Virus (HIV) plus breech presentation, preeclampsia plus latent phase of labour and elective caesarean section were excluded because the partograph completion is not recommended for mothers with the aforementioned characteristics.

### Sampling method

The sample size in the present descriptive retrospective document review was determined using a single proportion formula $n\;=\;\frac{Z_{\left(\alpha /2\right)}^{2}p\left(1-p\right)}{w^{2}}$ where n is the required sample size, **z** is the standard normal deviate, set at 1.96 (for 95% confidence level), **w** is the desired degree of accuracy (taken as 0.05) and **p** is the estimate of the proportion of the modified WHO partographs on which all components of the partograph are recorded up to the standard (assumed to be 24% as obtained from a retrospective study done in Kenya on the use of partographs in public health facilities (Mugerwa KY, Namagembe I., Ononge S., Omoni G., Mwuiva M., Wasiche J., Masbayi V, (2008) “unpublished observations”). Thus, the sample size required for this study was estimated to be 280 partographs. Due to multistage nature of the study, a design effect of **1.5** was considered. Hence, **n = [1.96**^**2**^ **× 0.24(0.76)/0.05**^**2**^**] × 1.5 =420.** Accordingly, the final sample size was **420** modified WHO partographs.

A multi-stage sampling technique was employed to select the partographs required for this study. From all maternity service provider public health institutions (25 health centres and 5 hospitals), two hospitals and three health centres were selected by simple random sampling technique. Three consecutive months of the year 2011 and 2012, namely December 2011, January 2012 and February 2012 were selected for record review because of the fact that they provided adequate and current information about partograph completion in the selected institutions. The sample size of 420 partographs was allocated to the institutions included in the study proportional to the number of the modified WHO partographs in the maternity units of the respective health institutions. Monthly records of number of deliveries (Average number of deliveries for the three consecutive months) were used in each institution for this purpose. At each study center, systematic sampling was employed to select the proportionally distributed samples starting from the latest month backwards until the required sample size was reached.

### Data collection

A pre-tested and structured checklist was developed, after reviewing literatures relevant to the problem under study, to include most of the possible variables that address the objectives of the study. The checklist was designed to obtain information on the main variables included as components of the modified WHO partograph. In order to produce a more objective assessment, the parameters of labour/parts of the modified WHO partograph were assessed to determine whether they had been monitored according to standard protocol [9]. Standard protocols were defined based on the time interval as follows:- (1) cervical dilatation, moulding, descent of the presenting part and blood pressure monitored every four hours; (2) foetal heart rate, maternal pulse and uterine contractions monitored every 30 minutes; (3) Condition of the baby after birth should always be recorded on the card. Records not meeting any one of the protocol standards or with parts misplaced/missing or inadequate for each parameter of the partograph were judged as **substandard**, or **not recorded** if no information was documented on the parameters of the partograph or completely absent from the file and **standard** if all the criteria are met for each parameter on the partograph. The condition of the baby should also have been recorded in appropriate section of the partograph to include the Apgar score (Apgar score of ≥7 was considered satisfactory in this study) [10].

A team of data collectors, including one of the authors (EY), systematically reviewed all the partographs and documented the required information using the checklist. All partographs were scrutinized for documentation of cervical dilatation, uterine contraction, foetal heart rate, action line crossed/not crossed, maternal blood pressure, moulding, descent of the presenting part, state of membranes and condition of the baby after birth.

### Data analysis

Data entry was performed using the software Epi Info Version 3.5.1. Data cleaning was done via a record screen of Epi Info using the *list* and *find* commands and the *sort* button and by cross-checking with the hard-copy checklist. The data were then exported to SPSS version 16 for further analysis. Frequency distributions, cross-tabulations and a graph were used to describe the variables of the study.

### Ethical clearance

As this study was based on document review, it didn't involve direct contact with human study participants. Thus, it didn't have serious ethical issues. Yet, during data extraction from the documents selected for the study, names of clients/patients to whom the documents belonged were not taken and as such the data remained anonymous. Besides, the study was conducted after obtaining ethical clearance from the ethical review committee of the Department of Nursing and Midwifery of Addis Ababa University and from the ethical review committee of Addis Ababa Regional Health Bureau.

## Results

Four hundred and twenty of the modified WHO partographs that had been used for labour management in five health facilities during the period of this study were reviewed. The number of deliveries from December 2011 to February 2012 (document review months) ranged from 87 (at Lideta health centre) to 1660 (at Gandhi memorial hospital). There were no written guidelines on how to complete the partograph for recording and management of labour in any of the labour wards.

The five health facilities included in the present study are presented in Table 1.

**Table 1:** Public health facilities included in the study, Addis Ababa, 2012

**Types of institutions**	**Name of institutions**	**Partographs selected**	
		**n**	**%**
**Hospitals**	Gandhi memorial hospital	234	55.7
	Yekatit 12 hospital	114	27.1
**Health centres**	Gulele health centre	27	6.4
	Kotebe health centre	28	6.7
	Lideta health centre	17	4.0
**Total**		420	100.0

Based upon review of 420 of the modified WHO partographs across all the health units, foetal heart rate was not recorded in 174 (41.1%) and the records were judged to be sub-standard in 117(27.9%) while recorded up to the recommended standard in 129(30.7%) of the partographs reviewed.

In 364 (86.7%) of the 420 modified WHO partographs reviewed, moulding of foetal head was not recorded at all, while in 26 (6.2%) and in 30 (7.1%), it was plotted below the standard and up to the recommended standard respectively.

The status of membranes was recorded only in 113 (26.9%) of the partographs reviewed while not recorded at all in 307 (73.1%) (See Table 2).

**Table 2:** Recording of parameters of feotal wellbeing, public health institutions of Addis Ababa, December 2011—February 2012

**Parameters of labour**	**Frequency (n = 420)**	**%**
**Feotal heart rate**		
Not recorded	174	41.4
Substandard	117	27.9
Monitored to Standard	129	30.7
**Moulding**		
Not recorded	364	86.7
Substandard	26	6.2
Monitored to Standard	30	7.1
**Was the status of membranes recorded?**		
Yes	113	26.9
No	307	73.1

Measurement of cervical dilatation was recorded in 248(59.1%) of the partographs but almost half [110(44.4%)] of these records were substandard while cervical dilatation was not recorded in 172(41.4%) of the partographs. Uterine contraction was not recorded in 189 (45.0%) while recorded to the standard in 87(20.7%) and sub-optimally recorded in 144 (34.3%) of the partographs.

Descent of the presenting part was not recorded in 353(84.0%) of the partographs reviewed. The action line of the cervical graph was crossed only in 15(10.9%) of the recorded partographs. Two hundred seventeen (51.7%) deliveries during the period of study had their blood pressure monitored. Seventy eight (18.6%) were monitored to standard while 139(33.1%) were substandard. Condition of the baby after birth was assessed using the Apgar score system. Apgar score was not recorded in 17 (4.1%) of the studied partographs. In 79.3% of the partographs, where the condition of the new born had been recorded, live babies were considered to have been born in good condition (Apgar score 7–10) (Table 3).

**Table 3:** Recording of parameters of maternal and feotal conditions, public health institutions of Addis Ababa, December 2011—February 2012

**Parameters of labour**	**Frequency (n = 420)**	**%**
**Descent of foetal head**		
Not recorded	353	84.0
Substandard	38	9.0
Monitored to Standard	29	6.9
**Cervical dilatation**		
Not recorded	172	41.0
Substandard	110	26.2
Monitored to Standard	138	32.9
**Uterine contraction**		
Not recorded	189	45.0
Substandard	144	34.3
Monitored to Standard	87	20.7
**Action line crossed (n = 138)**		
Yes	15	10.9
No	123	89.1
**Blood pressure**		
Not recorded	203	48.3
Substandard	139	33.1
Monitored to Standard	78	18.6
**Condition of the baby after birth**		
Not recorded	17	4.0
**Recorded**		
Good (Apgar score 7–10)	333	79.3
Not good (Apgar score 1–6)	57	13.6
Still birth	13	3.1

**Figure 2:**
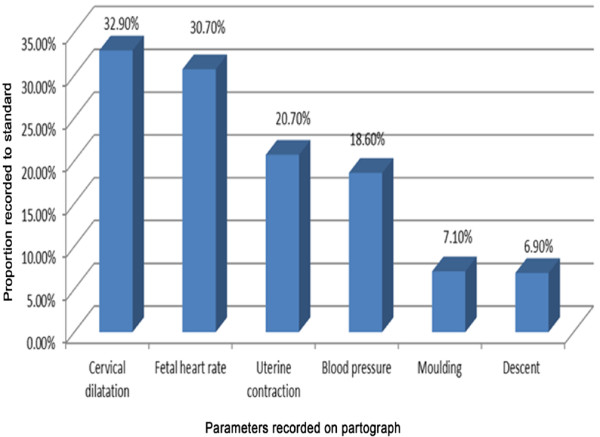
Proportions of partographs on which parameters were recorded to standard, public health institutions of Addis Ababa, December 2011—February 2012.

From labour parameters monitored on the reviewed partographs, cervical dilation was better monitored (32.90%). The least standard monitored parameter was descent of the feotal head which was only recorded in 6.9% of the partographs (See Figure 2).

## Discussion

The present study revealed high proportions of unrecorded parameters of labour on the modified WHO partograph and substandard monitoring of the progress of labour among public health institutions in Addis Ababa, Ethiopia. Lack of records for the descent of the presenting part in 84%, moulding in 86.7% and the foetal heart rate in 41.1% of the studied partographs indicates poor documentation, and perhaps monitoring and supervision of labour. In order to achieve good foetal outcome, it is extremely important to monitor foetal condition during labour [11]. It was hoped that completion of this instrument would help towards achievement of that goal.

The present study also found that all health units did have the modified WHO partographs available but were not completing them properly. Among 420 of the modified WHO partographs reviewed across all the health units, foetal heart rate was not recorded in 174 (41.1%) and was sub-standard in 117(27.9%) while monitored up to the recommended standard in 129(30.7%) of the partographs. This finding reported a higher figure than a study done in Uganda [10] where the partograph documentation that fulfilled the standard monitoring of foetal heart rate was only 2%. This difference could be due to differences in the health system obligatory policy on the use of a partograph during labour and the time gap between the present study and the study in Uganda which was conducted from May 23rd to 27th June, 2008 [10].

In this study only 32.9%, 30.70% and 20.70% of the foetal heart rate, cervical dilation and uterine contraction respectively were recorded according to the standard for monitoring of these three labour parameters. This is indicative of poor monitoring of parameters on the partograph against standards. The findings of similar studies done in Tanzania, Uganda and Benin [9,10,12] also showed poor monitoring of the parameters of labour against the accepted standards. This necessitates the need for regular pre-service and on-job training of obstetric care givers on completion of the partograph and perhaps a mandatory health facility policy on the completion of the partograph.

Similar to study reports from Tanzania and Uganda [9,10], cervical dilation was relatively more frequently (32.9%) recorded to the recommended standard, while uterine contraction was not recorded in 45% of the partographs reviewed. This is similar to the study reported from Dar es Salaam, Tanzania [9] where cervical dilation was the most frequently recorded parameter of the progress of labour (up to 97%), while uterine contraction was not recorded in almost two thirds (61%) of the partographs reviewed. Such a wide variation in the records may suggest that obstetric care givers prioritized documentation of cervical dilation over the other parameters.

This study has further revealed that the majority of the obstetric care givers had few skills on the appropriate completion of the partograph as all of the labour parameters were recorded to standard in less than 40% of the modified WHO partographs reviewed. This finding was slightly lower than the findings from Dar es Salaam, Tanzania [9] where only two labour parameters were monitored by over 40% of the partographs.

Lack of documentation and suboptimal documentation of some parameters of the progress of labour could hinder early detection of complications. Early detection and timely intervention on obstetric complications are the most important activities to prevent maternal and perinatal mortality and morbidity [13]. The poor documentation of the parameters found in the present study likely reflects poor intrapartum care and could partly explain the existing high maternal and perinatal mortality in Ethiopia [14,15]. A pre and post educational assessment along with the documentation of outcomes may provide further impetus for appropriate completion of the partograph.

The limitations of this study could include the following. Firstly, the study assessed only the completion of the parameters of the partograph during labour. As completion may not necessarily mean use, the findings of the present study may not show the extent of use of the partograph for monitoring labour progress. The partographs might have been used only to record events in labour rather than to guide management of labour. Secondly, the study could not assess documentation of results of biochemical tests on the modified WHO partographs like tests for urine sugar as these were expected to have been rarely performed due to non-availability of required supplies in all units. Lastly, as this study is confined to public health facilities of Addis Ababa, Ethiopia, the findings may not be generalizable to private health facilities as well as to public and private health facilities out of Addis Ababa.

In conclusion, the present study showed a poor completion of the modified WHO partographs during labour in public health institutions of Addis Ababa, Ethiopia. The findings may reflect poor management of labour or simply inappropriate completion of the instrument.

Based on the findings of this study, and supportive of earlier recommendation [8], pre-service and periodic on-job training of health workers on the completion of the partograph, regular supportive supervision, provision of guidelines and mandatory health facility policy are recommended.

## Abbreviations

AARHB: Addis Ababa regional health bureau; HIV: Human immunodeficiency virus; IUFD: Intrauterine foetal death; NGOs: Non-governmental organizations; WHO: World health organization.

## Competing interests

The authors declare that they have no competing interests.

## Authors’ contributions

All authors (EY, BD, AA, and NF) contributed to the design of the study and the interpretation of data. EY performed the data analysis and drafted the manuscript. All other authors (BD, AA,NF) critically revised the draft manuscript. All authors read and approved the final manuscript.
